# An integrated network visualization framework towards metabolic engineering applications

**DOI:** 10.1186/s12859-014-0420-0

**Published:** 2014-12-30

**Authors:** Alberto Noronha, Paulo Vilaça, Miguel Rocha

**Affiliations:** Centre of Biological Engineering (CEB), School of Engineering, University of Minho, Campus de Gualtar, Braga, Portugal; SilicoLife, Lda, Braga, Portugal

**Keywords:** Metabolic network visualization, Metabolic engineering, Open-source software

## Abstract

**Background:**

Over the last years, several methods for the phenotype simulation of microorganisms, under specified genetic and environmental conditions have been proposed, in the context of Metabolic Engineering (ME). These methods provided insight on the functioning of microbial metabolism and played a key role in the design of genetic modifications that can lead to strains of industrial interest. On the other hand, in the context of Systems Biology research, biological network visualization has reinforced its role as a core tool in understanding biological processes. However, it has been scarcely used to foster ME related methods, in spite of the acknowledged potential.

**Results:**

In this work, an open-source software that aims to fill the gap between ME and metabolic network visualization is proposed, in the form of a plugin to the OptFlux ME platform. The framework is based on an abstract layer, where the network is represented as a bipartite graph containing minimal information about the underlying entities and their desired relative placement. The framework provides input/output support for networks specified in standard formats, such as XGMML, SBGN or SBML, providing a connection to genome-scale metabolic models. An user-interface makes it possible to edit, manipulate and query nodes in the network, providing tools to visualize diverse effects, including visual filters and aspect changing (e.g. colors, shapes and sizes). These tools are particularly interesting for ME, since they allow overlaying phenotype simulation results or elementary flux modes over the networks.

**Conclusions:**

The framework and its source code are freely available, together with documentation and other resources, being illustrated with well documented case studies.

**Electronic supplementary material:**

The online version of this article (doi:10.1186/s12859-014-0420-0) contains supplementary material, which is available to authorized users.

## Background

Within the field of Systems Biology, the analysis of different types of biological networks is an important task in understanding the underlying biological processes. For this endeavour, a mathematical framework is provided by graph theory, which has allowed to verify that a multitude of organisms share relevant properties when analysing the topology of their networks [1]. Additionally, being able to capture the network in a visual form can provide useful insights. While, in the pre-genomic era, the analysis and visualization of these networks were approached as independent computational problems, it is desirable that these two levels are well integrated [2].

Together with their regulatory [3] and signalling counterparts [4,5], metabolic networks represent a vastly studied class of biological networks. These are typically composed of two entities: metabolites and reactions. Metabolites can be converted by the cell into building blocks or decomposed to generate energy or other compounds.

Metabolic Engineering (ME) aims to rationally pinpoint genetic changes in selected host microbes that can optimize the production of compounds of industrial interest and, thus, it heavily makes use of computational analyses of metabolic networks. However, in many cases, the static view of metabolic systems provided by these networks is insufficient, and there is the need to reconstruct genome scale metabolic models (GSMMs) [6] with simulation capabilities, which are increasingly being created given the availability of genome sequences, annotation tools and omics data.

Given the lack of kinetic information to provide for large-scale dynamical models, stoichiometric models are the most common. The information about the metabolites and reactions from the metabolic network, together with stoichiometry, are the starting points for their reconstruction [7], being mathematically represented by a set of equations that describe the chemical transformations [8].

GSMMs are often used to simulate the metabolism of the cell using constraint-based approaches, where typically a pseudo steady state is assumed [9]. Using these models and specifying environmental conditions (e.g. media), it is possible to perform the calculation of flux distributions. The most used method is Flux Balance Analysis (FBA), where a flux (e.g. a biomass flux) to maximize (or minimize) is chosen to obtain an optimal flux distribution [10].

In this work, an integration of network visualization with ME methods is proposed, which demands that many issues related with the visualization of metabolic networks must be addressed. While the scalability of the networks is successfully addressed by generic visualization packages, usually the generic layouts available produce unsatisfactory results for metabolic networks. This is mostly due to the fact that the majority of layout algorithms do not take into consideration any biological knowledge, such as cell localization or molecular functions. Another problem comes with the filtering of the networks. It is necessary to have an easy way to query the network and visually filtering it to specific sets of nodes of interest in a given context. To address these problems, a visualization tool should offer some basic features: layout algorithms, a graphical notation, integration with analysis tools by providing information about the network and an user interface to allow interaction [7].

There are a myriad of software tools for ME, able to use metabolic models to perform phenotype simulations and implement strain optimization methods, being some of the best known examples: *OptFlux* [11], the *COBRA Toolbox* [12,13], *CellNetAnalyzer* [14] and *FASIMU* [15]. There are also several tools that perform visualization of metabolic (and other types) of biological networks. There are not, however, many examples of successful integration of these two distinct types of applications. *CellDesigner* [16,17], for instance, is one of the most popular tools for editing and visualizing biochemical networks, but it lacks specific methods for constraint-based approaches and does not deal well with large-scale GSMMs.

*Cytoscape* [18] became a standard tool for the integrated analysis and visualization of biological networks. One of its many plugins, *FluxViz* [19], provides features for the visualization of flux distributions in networks. *FluxViz* was primarily developed for *FASIMU*, a software for flux-balance computation, and it uses the generated result files as input for visualization.

Another tool worth mentioning is *VANTED* [20], an application for the visualization and analysis of networks with related experimental data. The usefulness of this tool for ME purposes is provided by two of the available plugins, *FluxMap*, that allows the visualization of measured or simulated fluxes in the network, and *FBASimVis* for constraint-based analysis of metabolic models, with a special focus on the dynamics and visual exploration of metabolic flux data resulting from model analysis. It supports wild type and knock-out FBA simulations.

Both the *COBRA toolbox* and *CellNetAnalyzer* are based on the commercial software *Matlab*, and therefore are not freely available for the community. *COBRA* already includes some network visualization tools in the original release, but the generated maps are static built-in maps, that can be exported in a single format (SVG), optionally including overlaps with simulation results. An extension, Paint4Net [21], that allows some editing features (mainly node dragging), has been recently proposed. However, editing is limited to the models and layouts from the related *BiGG* database, editing options are quite limited and layouts can only be exported as images and not reused. On the other hand, *CellNetAnalyzer* only enables the visualization of small or medium scale biological networks. More recently, *MetDraw* [22], that is capable of generating layouts for large metabolic models, was published providing the means to visualize “omics” data overlaid in the network. However, it does not support editing layouts, requiring the use of an external tool for that aim, and is not integrated with any ME tool.

In the majority of these tools, biological entities/ interactions are represented as shapes/ lines, with different colours/formats standing for their classes. Although this seems a reasonable solution, the complexity of the integrated information and the range of possible interactions motivated the development of standard notations. The most successful was the Systems Biology Graphical Notation (SBGN) [23], where networks are modelled in a state-transition way. Another successful standard format is the Systems Biology Markup Language (SBML) [24], which aims at storing and exchanging biological models. Combined with the development of the SMBL Layout package, this makes up a very promising effort in network visualization as well. The full support to these standards is not guaranteed by most of the tools and this would be an advantage in the interoperability of these tools with other relevant software.

Overall, and in spite of the aforementioned tools, network visualization has been traditionally apart from ME-related methods. Some notable exceptions were already mentioned, but an effective framework, which would facilitate the agile integration of simulation results with dynamic layouts of metabolic networks, is, in the authors’ point of view, still lacking. Indeed, this work focuses on the development of a visualization framework based on a well-defined abstract representation of metabolic networks, which will provide researchers with visualization tools, to be used in the context of ME projects. This has been developed as a plugin for the *OptFlux* platform, allowing its integration with other tools, building a useful tool to assist ME researchers. To highlight the main features of the tool, described in detail next, as compared to the other tools, Table 1 provides a comparative analysis of their features.

**Table 1:** **Feature comparison of several tools for metabolic network visualization**

	**OptFlux visualization plugin**	***CellDesigner***	**Cytoscape**	**VANTED**	**Paint4Net**	**CellNet-analyzer**	***Cobra toolbox***
**File formats/standards**							
SBML	●	●	●	●	●	●	
SBGN	●	●	●				
GML			●	●			
XGMML	●		●				
KGML	●		●	●		●	
Cobra maps	●						●
**Layout algorithms**							
FDL	●		●	●	●		
Hierarchical		●	●		●		
Circular		●	●	●			
Grid			●	●			
Organic		●	●				
**Integration with ME**							
Flux distributions	●		●	●	●	●	●
Genetic conditions	●					●	
Elementary flux modes	●					●	
Simulation comparison	●			●		●	
**Other features**							
Layout generation	●	●	●	●	●	●	
Layout exportation	●	●	●	●	●	●	●
Edition	●	●	●	●	●	●	
Multiple layouts	●	●	●				
Partial layouts	●	●	●	●	●	●	●

## Implementation

Metabolism can be represented as a series of transformations of metabolites, being easy to represent as a graph. There are two main entities that will be addressed by the visualization platform: reactions and metabolites. A reaction is a chemical transformation that uses a set of metabolites as reactants and produces another set of metabolites to be used by other reactions.

**Figure 1:**
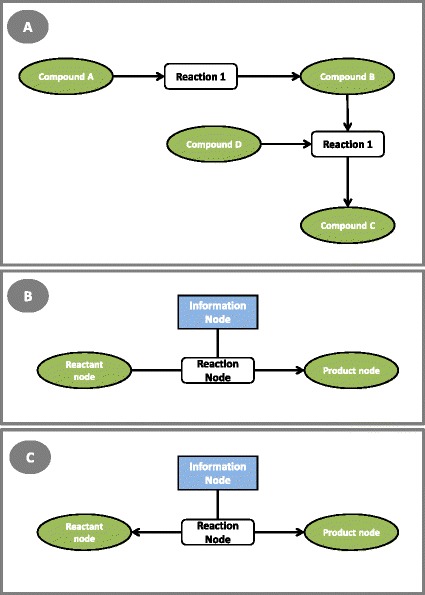
**Network representation used in the visualization framework. A** reaction-compound network; **B** adopted irreversible reaction representation; **C** adopted reversible reaction representation.

For the representation, a reaction-compound network (Figure 1A) was chosen, represented by a bipartite graph, which can be divided into two distinct sets of vertices (nodes), such that every element of a set only connects with vertices of the other set. This provides a descriptive and visually attractive representation.

A metabolic layout can then be defined by a core list of reactions, each represented by a reaction node, a set of reactants, a set of products and a set of information nodes. The reactants and products are represented as sets of metabolite nodes, representing the compounds that are part of that specific chemical reaction. The reaction node, and the respective metabolite nodes, will be connected by edges, represented as lines with a shape defined according to the reversibility of the reaction. If the reaction is irreversible, the edges that connect reactions to the metabolites will have arrows only pointing to the products (Figure 1B), while in reversible reactions they will have arrow shapes pointing to both metabolite ends (Figure 1C). The metabolite nodes can have two distinct types: regular and currency metabolites. The ones in the latter group will be differentiated since they typically represent highly connected hubs (e.g. water, co-factors) with reduced interest in most analyses.

A metabolic layout is based on the reactions contained in the metabolic model. Since one of the goals of this work is to provide a link between the visualization and the metabolic model, a strategy must be defined to map the entities of the visualization framework with the entities of the model. It is also desirable that a layout can represent just a part of the metabolism of an organism (allowing different layouts for the same model), as well as the possibility to use the same layout on different models (e.g. different strains or model versions). Another important aspect is to make networks visually more understandable, replicating some of the nodes, a feature typically applied to currency metabolites. To comply with these features, each reaction and metabolite node will have a list of model identifiers that will provide the link between the model and the layout.

**Figure 2:**
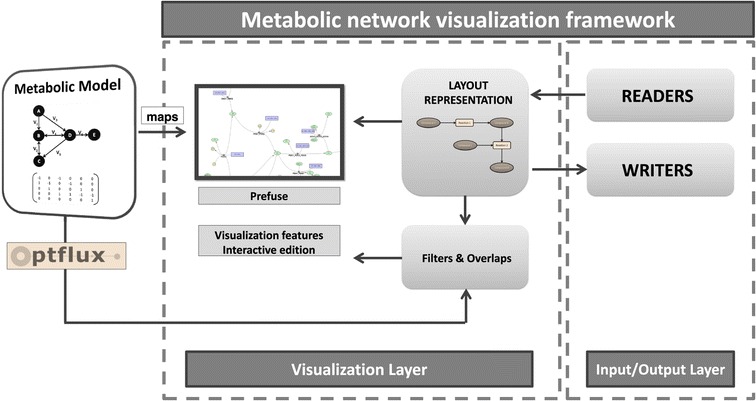
**Visualization framework architecture overview.** The Input/Output layer provides the reading and writing capabilities for several file formats. The visualization layer contains the layout representation, and provides the visualization capabilities. External sources can also provide filters and overlaps, being OptFlux one such example (through the visualization plugin).

The two main tasks of the software are to build these layouts from external sources (being able to export them as well), and to visually represent them. This leads to a two-layer architecture (Figure 2), where the first implements the capabilities to read and write metabolic layouts, while the other, the visualization layer, handles the visualization and edition of the metabolic layout. The main features of each are given below:

- **Visualization layer**: provides all the functionalities related with the visualization and edition of a layout, including automatic layouts, creation and edition of layouts, visual filters and operations to change the aspect of the network (colours, shapes, etc.).
- **Input and output layer**: implements several tools that provide network creation and exportation capabilities for a multitude of file formats. It has the objective of reading networks in specific file formats and building the metabolic layout, used by the visualization layer. At the same time, it also provides the possibility to export those layouts into some of those formats.

The strategy adopted in the development of the framework had the goal of creating a tool that can be used independently, but at the same time build it in a way such that the integration with an ME tool (*OptFlux* in this case) was facilitated. This brings to light the importance of the MVC (model-view-controller) design pattern, not only in the development of the framework, but also in the integration with *OptFlux*, that also follows this principle. Indeed, *OptFlux* is built on top of *AIBench* (http://www.aibench.org/), a software development framework developed by researchers from the University of Vigo in Spain. Building applications over *AIBench* facilitates the creation of applications composed of units of work with high coherence that can easily be combined and reused, by incorporating three main object types: *operations*, *datatypes* and datatype *views*.

The basis of the implementation of the operations in *OptFlux*, is a core library implementing relevant ME methods and algorithms, including phenotype simulation methods (e.g. FBA, parsimonious FBA, MOMA and ROOM), strain optimization algorithms and with many other features. It also contains all data structures and methods used to represent metabolic models, and reading/writing files in different formats. *OptFlux’s* plugin-based architecture facilitates the development of additional features. The visualization plugin is such an example, which provides a direct connection between the metabolic model, simulation and optimization methods used in *OptFlux,* and the metabolic layout defined in the visualization core framework.

The entire visualization framework was developed in Java. The visualization of the graph is done using the *prefuse* package (http://prefuse.org/), the CD-SBML is read using *JSBML* [25], SBGN-ML files are read using *libSBGN* [26] and the exportation of the layouts as images (SVG and PDF formats) is performed using *Batik Java SVG Toolkit* (http://xmlgraphics.apache.org/batik/) and *VectorGraphics2D* (http://trac.erichseifert.de/vectorgraphics2d/).

## Results and discussion

### Visualization layer features

**Figure 3:**
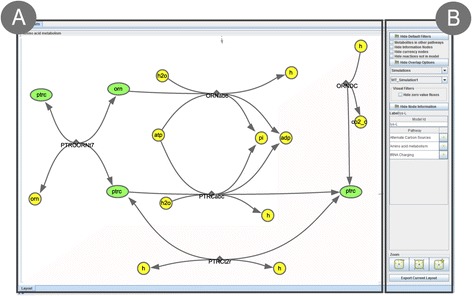
**Visualization framework interface examples. A** network view where the network is displayed, and the user can interact with it; **B** side panel where filters, overlaps, information about the nodes and the zoom panel are displayed.

As stated previously, the visualization layer provides all the functionalities related with the visualization and editing of the metabolic layout. One of these features allows to change the default colours and shapes of the nodes. The graphical user interface (GUI) is composed of two major elements (Figure 3): the network view, where it is possible to edit the network and click/drag the nodes (Figure 3A), and the side panel where filters, overlaps and node information are available (Figure 3B). In this way, it is possible for the user to easily interact with the network, using all the features the interface has to offer.

This layer provides all the features that allow navigating and obtaining additional information about the network by clicking the nodes. Some of them are the basic features that are typical in any visualization framework, such as highlighting, zooming and dragging, which allow navigating through the network. By clicking a node, the node information panel will display information on that node. It is possible for advanced users to implement an information panel and add it to the interface, to visualize information of specific interest.

One of the main features allows loading layouts from different sources. Some layouts may not specify the coordinates of (some of) the nodes. The layout used by the visualizer, by default, is the *Force Directed Layout* (FDL) [27] with some modifications to support fixed nodes. This was coupled with the possibility to fix/unfix nodes, allowing the user to fix a node to the specific position it is in, or drag it to a desired position; unfixing a node will remove the position information of the node, making it susceptible to the FDL algorithm that can adjust its position according to its surroundings. It is also possible to unfix and fix nodes by type, allowing a user to fix/unfix all reaction or metabolite nodes at the same time.

Another crucial aspect is the ability to edit the metabolic layout. This feature, when combined with the import and export capabilities, provides users with the means to create and edit their layouts, being able to export them for later use.

As stated above, the same metabolite can be represented several times in a layout by different nodes. If a metabolite node is connected by several reactions the user can replicate it, resulting in a metabolite node for each reaction. Also, metabolite nodes representing the same compound can be merged. It is also possible to replicate a reaction node, resulting in two, or more, reactions connected to the same set of metabolite nodes. Merging two reactions is only possible if they are exactly the same, i.e. are connected to the same nodes and have the same reversibility. The type of a metabolite can be changed, e.g. to a currency metabolite.

Filtering and overlaying capabilities are also provided. It is possible to filter the network, by hiding parts of it, based in the node type (e.g. hide all currency metabolites) or by reaction identifier. To overlay information over the network, the visualizer allows altering its visual aspect, supporting the change of the direction, thickness and colours of the edges, while for nodes it is possible to change the colour and shape. This feature will allow, for instance, overlaying flux distributions in the metabolic layouts.

### Input and output features

The input/output layer provides support for different input/output formats:

- **CellDesigner SBML (CD-SBML)**: a graphical notation system proposed by Kitano [28], where layouts are stored using a specific extension of the Systems Biology Markup Language (SBML).
- **eXtensible Graph Markup and Modeling Language (XGMML)**: a format based on the Graph Modelling Language (GML), being used for graph description using XML tags to describe nodes and edges of a graph. It is used, for instance, by *Cytoscape*.
- **KEGG Markup Language (KGML)**: an exchange format created for the automatic drawing of Kyoto encyclopaedia of genes and genomes (KEGG) [29] maps.
- **Systems Biology Graphical Notation (SBGN)**: defines three visual languages: Process Description (PD), Entity Relationship (ER) and Activity Flow (AF). For the purpose of this work, which focuses on metabolism, support was only developed for the PD language, based on Kitano’s proposal used in *CellDesigner*’s graphical representation, using bipartite graphs.
- **COBRA Layouts**: maps developed for the *COBRA Toolbox*. There are, currently, several maps on this format for many of the models hosted in the BiGG knowledgebase (http://bigg.ucsd.edu). These can be used on different models that have similar pathways, with a correct mapping of the identifiers between the layout and the BiGG model.
- **Pathway generation**: It is possible to generate a layout by using a list of reactions from a GSMM. This can be done following two strategies: choosing a list of reactions or, in the case where the model has pathway information, building layouts with the reactions from a set of pathways.

### OptFlux plugin

The visualization plugin for *OptFlux* has the main goal of providing a connection between the GSMMs loaded into *OptFlux,* their phenotype simulation and optimization results, and the layouts from the visualization framework.

Through the plugin’s operations, it is possible for the user to map the identifiers of the metabolic model with the identifiers of the reaction and metabolite nodes of the layout. There are two different mapping methods available: loading a two-column file with the explicit mapping or applying regular expressions to the identifiers in the model and/or the layout. Another available operation allows the importation of KGML layouts, which can be automatically downloaded from the KEGG site.

The third operation allows the creation of layouts from reactions of a metabolic model, using the pathway layout generation feature described above. The generation of this type of layouts can be made by selecting a pathway from the model or by selecting a list of reactions manually. It is also possible to select an existing layout as a basis for the new layout. This will allow creating new layouts or adding new reactions to existing ones.

Each model can have a list of layouts associated, being possible to navigate from one layout to another by clicking the elements of that list. If the user clicks a metabolite that is present in another layout from the list, the information panel will display access buttons for those layouts.

The most desired functionality of the connection between a ME and a visualization tool, is the ability to visualize phenotype simulation results (mainly flux distributions) overlaid in the network. This allows using the visualization tool to better understand the organism’s metabolism and design changes that can improve it towards some defined aim.

To allow this operation, there is a conversion from a simulation result in *OptFlux*, to an overlap object that is used in the visualization. In *OptFlux*, simulation results have two major elements of interest for the visualization: flux distributions and genetic conditions. A flux distribution contains the flux values for each reaction. To represent it, a conversion of identifiers is needed. It can happen that two or more fluxes are mapped to the same reaction node, and the methodology chosen was to sum all those values (although alternative options can be easily implemented). In the end, all these flux values, now mapped by reaction node, are normalized and used to determine the thickness of the edges. Additionally, the labels of the reaction nodes are also changed, adding the numerical value of the flux after the reaction name.

**Figure 4:**
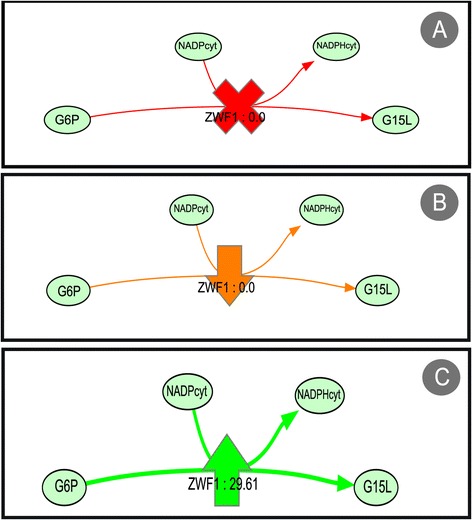
**Symbols used to represent genetic modifications in a phenotype simulation overlap. A** knocked-out reaction; **B** under-expressed reaction; **C** over-expressed reaction.

The genetic conditions of a simulation are defined as all genetic changes made to the organism for that specific simulation. It contains all knock-outs (reaction deletions), and under/over expressed reactions. For the visual representation, some node shapes and colours were adopted to highlight these affected reactions. As seen in Figure 4A, a knocked-out reaction will be indicated by a red cross, with reaction edges also coloured red. An upward arrow will indicate an over-expressed reaction, where both the arrow and the edge are green (Figure 4B). Finally, an underexpressed reaction is coloured orange and accompanied by a downward orange arrow (Figure 4C).

Another type of overlap was also developed to visualize the comparison of two phenotype simulation results. The methodology followed was similar to the simulation overlaps. The genetic conditions are represented using the same symbols, but the colours of the edges follow a different strategy. Each simulation will have a colour by default, for instance, simulation 1 will have the colour red, and simulation 2 will have the colour green. Then, according to the flux for each reaction in each simulation, the colours will vary. If the amount of flux is larger in simulation 1, the colours will vary in a gradient that spans from red to black (where black means that there is no difference in fluxes), and if the flux value in simulation 2 is greater, the colours will vary from green to black. This will allow the user to identify where flux paths differ in the simulations (pure colours) and where both share fluxes (darker colours). At the same time, for reversible reactions, the fluxes of the compared simulations can take different directions. In this case, edges will have the colour of the simulation that follows the direction they are pointing, also giving the user an easy way to understand where the simulations differ. The thickness of the edges is calculated using the mean of the flux values in both simulations. On top of this, some filters are also generated, where it is possible to hide zero value fluxes in a simulation.

*OptFlux* also provides a plugin that calculates the set of Elementary Flux Modes (EFMs) of a model. EFMs are the set of all routes through the network that cannot be decomposed to simpler routes [30], while maintaining steady-state, so they provide a way to analyse the set of pathways in the metabolic network. This plugin provides an interface that allows filtering these results, including the selection of EFMs based on presence/absence of external metabolites or sorting by yield. It is possible to select sets of EFMs browsing these results, to visualize the EFMs in a column-wise table, and to obtain the flux values for each reaction within the EFM.

The visualization plugin can convert these flux distributions into an overlap, in a way similar to the one used for the phenotype simulation results. Considering that, in this case, the only information available is the set of flux distributions, only the thickness and labels of the edges are changed. A visual filter can be applied hiding the reactions with zero value fluxes, thus allowing the visualization of the reactions that are part of the EFM.

Regarding customization, the plug-in allows for the configuration of the style of the visualization through the Preferences option in the Help menu. This allows to personalize the layouts, defining parameters such as the colour and shape of the nodes or the font, size and colour of the labels. Perhaps more importantly, it is also possible to define the content of the labels of reaction and metabolite nodes, choosing which attributes to include from the ones available in the model. An example and more details of this process are given in the OptFlux documentation and in the case study description available in the Additional file 1.

### Usage example: succinate production with E. coli

To best illustrate the main features of the proposed tool, two case studies will be used, focusing on succinic acid (this section) and glycine production (next section) with *E. coli.* The full description of the workflow and required materials for both case studies are provided as Additional file 1.

Succinic acid is an important compound to industry that has been produced mainly by chemical processes. Recently, there has been an effort to use microbial fermentation processes with anaerobic bacteria [31] and optimizing micro-organisms to over-produce succinic acid is one goal of interest for ME researchers. For this case study, the *E. coli* metabolic model iJR904 [32] was used. The model is available for download directly from OptFlux’s internal repository, and it is composed of 1075 reactions (143 external and 932 internal), 761 metabolites (143 external, 618 internal) and 904 genes with 873 gene rules.

For visualization purposes, a COBRA layout was loaded for this particular model from the BiGG Database (http://bigg.ucsd.edu/). After some manual and automatic curation, using the tools offered by the visualization framework, a second version of the layout was created and exported in the XGMML format, fully mapped to the model. A set of knockouts was selected from optimization results obtained using *OptFlux* [33] (Table 2). This set was chosen because it is not composed of a large number of knockouts while, at the same time, it is somewhat complex.

**Table 2:** **Deleted reactions for succinic acid production with E. Coli, using the iJR904 metabolic model**

**Reaction**	**Model ID**	**Stoichiometric equation**
Serine hydroxymethyltransferase	R_GHMT2	L-serine + tetrahydrofolate < = > glycine + 5,10-methylenetetrahydrofolate + H_2_O
Pyridine nucleotide transhydrogenase	R_THD2	H^+^_e_ + NADH + NADP^+^ = > H^+^_c_ + NAD^+^ + NADPH
Succinate dehydrogenase	R_SUCD1i	FAD^+^ + Succinate = > FADH_2_ + Fumarate
Transkelotase I	R_TKT1	D-erythrose-4-phosphate + D-xylulose-5-phosphate < = > D-fructose-6-phosphate + D-glyceraldehyde-3-phosphate

**Figure 5:**
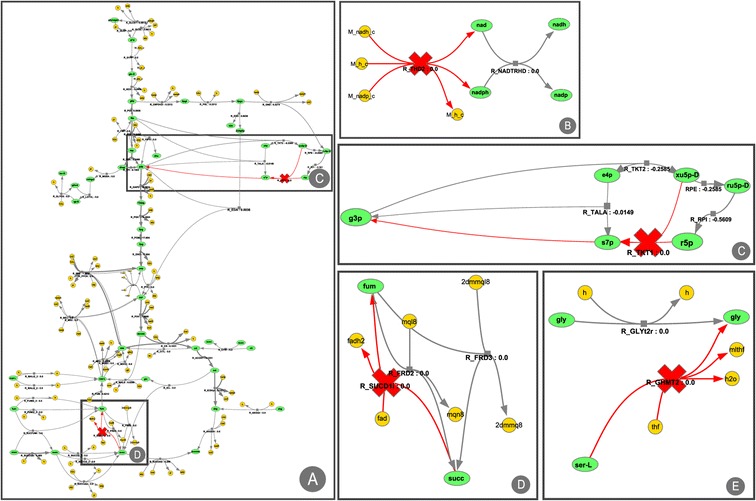
**Visualization of parts of the iJR904 model layout representing the main genetic modifications performed over the model to produce succinate. A** part of the central metabolism; **B** inactivation of the pyridine nucleotide transhydrogenase (R_THD2); **C** deletion of Transketolase I; **D** inactivation of the succinate dehydrogenase (R_SUCDli), in the TCA-cycle; **E** knockout of serine hydroxyl methyltransferase reaction (R_GHMT2).

In this case, the layouts used are of large dimensions so the full layout representation as an image is not practical. Figure 5 represents parts of the layout and important genetic modifications in the network.

By analysing the solution, and with the support from the visualization, it was possible to infer that NADPH balance was the key factor for the increase in succinate production. Figure 5A represents a part of the central metabolism of the *E. coli* iJR904 model. There, it is possible to visualize the deletion of Transketolase I (R_TKT1 – Figure 5C) that causes a decrease of flux in the pentose phosphate pathway leading to a decrease in NADH production. The inactivation of the pyridine nucleotide transhydrogenase (R_THD2 – Figure 5B) will also contribute to the shortage of NADH. The Serine hydroxyl methyltransferase reaction (R_GHMT2 – Figure 5E) is knocked-out to prevent the formation of NADPH in the glycine production pathway. Finally, to prevent the consumption of succinate, there is the inactivation of the succinate dehydrogenase, in the TCA cycle (Figure 5D), which leads to the excretion of succinate.

### Usage example: glycine production with E. coli

The second case study was performed with the iAF1260 *E. coli* metabolic model [34]. This model is larger than the one used in the previous example. It is composed of 2389 reactions (304 drains and 2085 internal), 1668 metabolites (304 external and 1364 internal) and 1260 genes. The goal of the optimization for this example was the production of glycine, and the results obtained are described in Table 3, chosen following the same strategy used in the previous case.

**Table 3:** **Deleted reactions for glycine production with E. Coli, using the iAF1260 metabolic model**

**Reaction**	**Model ID**	**Stoichiometric equation**
Isiocitrate lyase	R_ICL	Isocitrate = > Succinate + Glyoxylate
Glycine cleavage	R_GLYCL	Glycine + NAD^+^ + Tetrahydrofolate = > NH4^+^ + 5,10-Methylenetetrahydrofolate + NADH + CO_2_
Phosphoenolpyruvate carboxylase	R_PPC	Oxaloacetate + Phosphate < = > Phosphoenolpyruvate + Bicarbonate
Phosphoribosyglycinamide formyltrasferase 2	R_GART	5-phospho-ribosyl-glycineamide + Formate + ATP <= > 5’-phosphoribosyl-*N*-formylglycineamide + ADP + Phosphate + H^+^

For the visualization, several layouts were loaded containing most pathways of the metabolic model. Indeed, although it would be possible to load a layout for the entire model, this would not be practical to conduct the type of analyses shown below. In these cases, the user should seek to work with partial layouts with about 30 to 50 nodes (or up to 15 reactions) to improve the quality of the generated results.

**Figure 6:**
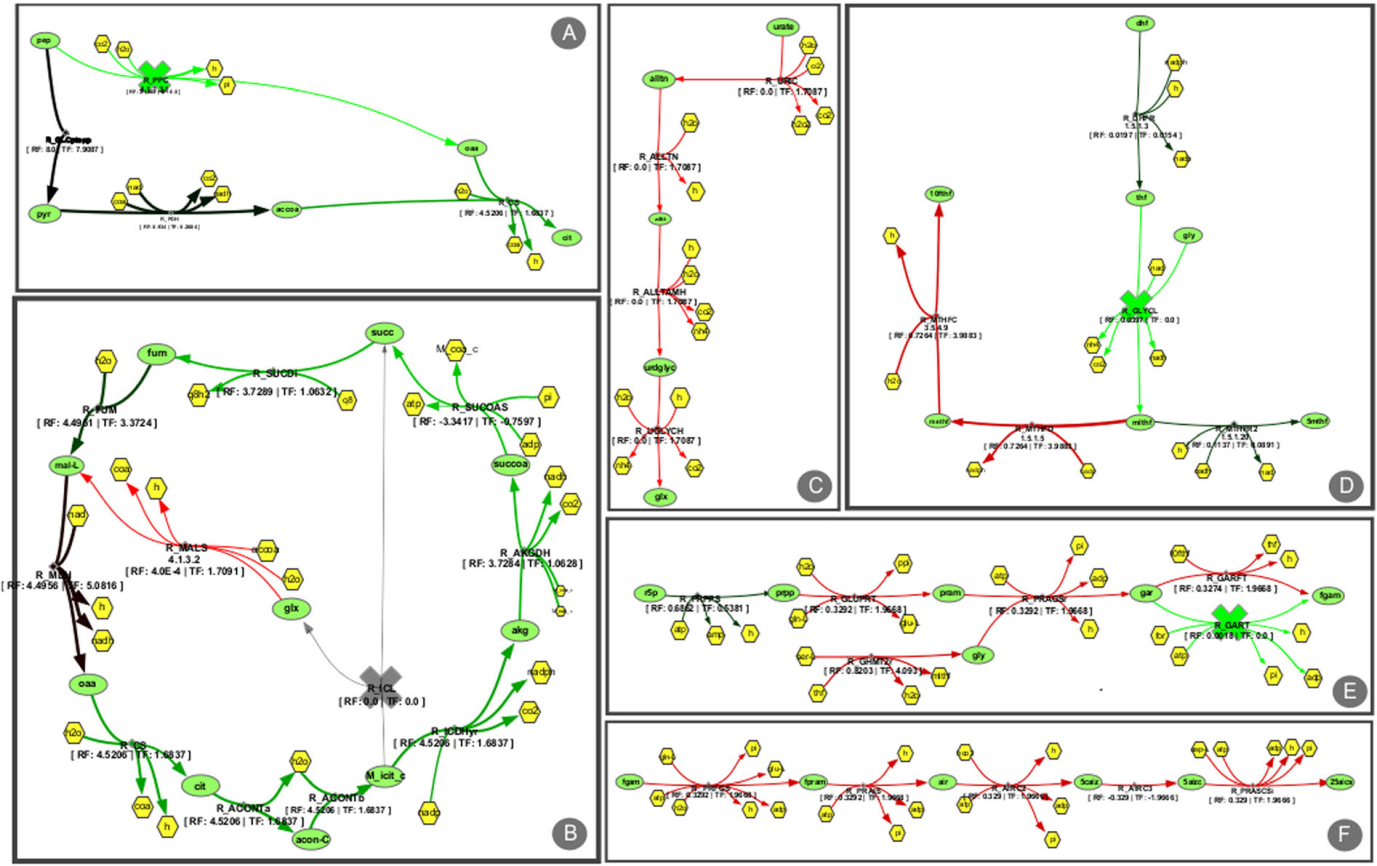
**Visualization of several parts of different pathway layouts of the iAF1260 model.** A simulation comparison of the wild-type simulation and a mutant that produces glycine, was performed, which allows the visualization of fluxes differences in both simulations. **A** part of central metabolism pathway, showing the inactivation of the phosphoenolpyruvate carboxylase (R_PPC); **B** another component of the central metabolism pathway showing the inactivation of the isocitrate lyase (R_ICL); **C** part of the alternate carbon souirces pathway that clearly shows an increase in flux for the mutant simulation. **D** part of the co-factor biosynthesis pathway showing the inactivation of the glycine cleavage (R_GLYCL); **E** and **F** parts of the nucleotide metabolism pathway and inactivation of the phosphoribosylglycinamide formyltransferase 2 (R_GART).

After loading the pathway layouts, a simulation comparison of the wild type and knock-out mutant was performed. Visualizing the overlap of that comparison, it is possible to understand how both flux distributions differ by analysing the colours of the edges. The reference flux distribution is the wild type, represented by the green colour, while the knock out solution will be coloured red. As explained above, the more similar both flux distributions are, the darker the resulting colours will be, ranging from black to the colour of the simulation with the higher value. Figure 6 shows parts of the layout and the relevant genetic modifications that lead to the optimized results.

The inactivation of Isocitrate lyase (R_ICL) and Phosphoenolpyruvate carboxylase (R_PPC), both present in the central metabolism (partially represented by Figure 6A and B), lead to the necessity of another route for the production of oxaloacetate. It is possible to see that the TCA cycle has mostly a prevalence of green colour, which means that the reference simulation, the wild-type, has more flux in those reactions. The other route taken due to these knockouts can be seen in two different pathways, being clearly visible as a chain of red reactions, both in the Alternate Carbon Sources layout (Figure 6C) and in the Nucelotide Metabolism (Figure 6E and F) culminating in the production of glyoxylate, which can be converted to L-malate by malate synthase (R_MALS – Central Metabolism Figure 6A) and then transformed into oxaloacetate. The inactivation of the glycine cleavage complex (R_GLYCL – Figure 6D) and phosphoribosylglycinamide formyltransferase 2 (R_GART – Figure 6E) are necessary to the accumulation of glycine, being produced as a by-product of *fprica* synthesis. Both reactions can be used to recycle glycine, which means that both deletions are essential to the solution.

## Conclusions

In this work, a metabolic network visualization framework was presented, which has the ability to load networks from a variety of formats and display them using a dynamic layout. It provides features for the straightforward creation and editing of these layouts, as well as exportation capabilities. On top of this, it is possible to overlay the network with visual changes, a functionality that allows, for instance, visualizing fluxes in a phenotype simulation, identifying the genetic conditions imposed in a simulation, addressing the comparison of two simulation results, analysing results from strain optimization methods or visualizing the set of elementary modes in a model.

The framework was integrated with *OptFlux*, a ME framework, by the development of a plugin. This allows ME researchers to use the visualization directly from within *OptFlux*, and use a series of operations that will allow loading and exporting layouts with a user-friendly interface.

This framework presents itself as a useful tool that can help researchers involved in ME projects to have a way of easily addressing the visualization of the metabolic networks they are studying. The ability to dynamically visualize phenotype simulations is an important asset. The combination of visualization with simulation and optimization processes will help researchers to achieve knowledge about the structure and functioning of organisms of interest that was not available before.

While a number of features are planned, an interesting line of future work is the development of tools that allow importing other types of omics data (e.g. gene expression or metabolomics), providing its integrated visualization with GSMMs. The general-purpose nature of the core layer of the visualization framework allows the easy development of such tools, providing a good basis for the extension of the proposed software also in other directions.

## Availability and requirements

The described plugin is included in the base distribution of *OptFlux* that can be downloaded and installed from the homepage given below. The site also includes documentation for the plugin in the form of a wiki.

More details:

- **Software name:** OptFlux - software for metabolic engineering
- **Project home page:**http://www.optflux.org
- **Specific wiki page for the plug-in (with help):**http://darwin.di.uminho.pt/optfluxwiki/index.php/OptFlux3:METAVIZ
- **Operating system(s):** Platform independent
- **Programming languages:** Java
- **Other requirements:** Java JRE 1.7.x (for MacOS the installation of Java SDK 7 is recommended), GLPK or CPLEX
- **License:** GNU-GPL, version 3

## Additional file

Additional file 1:
**Full description of the workflow followed in the case studies, including the full set of instructions to conduct them using** ***OptFlux.*** All materials needed for the tutorial are available on the URL: http://darwin.di.uminho.pt/optflux/suppmaterial/visualization/materials.zip.

## Abbreviations

CD-SBML: CellDesigner SBML
EFM: Elementary Flux Mode
FBA: Flux Balance Analysis
FDL: Force Directed Layout
GSSM: Scale metabolic model
GUI: Graphical User Interface
KEGG: Kyoto Encyclopedia of Genes and Genomes
KGML: Markup Language
ME: Metabolic Engineering
MOMA: Minimization of Metabolic Adjustment
MVC: Model-View-Controller
ROOM: Regulatory On/Off Minimization of metabolic flux changes
SBGN: Systems Biology Graphical Notation
SBML: Systems Biology Markup Language
XGMML: eXtensible Graph Markup and Modeling Language
