# Supplementary material for: An integrated network visualization framework towards metabolic engineering applications
Source: BMC Bioinformatics. 2014 Dec 30;15(1):420. doi: 10.1186/s12859-014-0420-0 (PMC4300605; doi:10.1186/s12859-014-0420-0)
Supplement: Additional file 1: — Full description of the workflow followed in the case studies, including the full set of instructions to conduct them using OptFlux. All materials needed for the tutorial are available on the URL: http://darwin.di.uminho.pt/optflux/suppmaterial/visualization/materials.zip. [file 12859_2014_420_MOESM1_ESM.pdf]

Detailed description of the case studies

Supplementary material for the paper: *An integrated network visualization framework towards metabolic engineering applications*

Introduction

To use Optflux's visualization plugin, you just need to download the most recent version of Optflux, as the plugin is already installed in the most recent versions. In this document some of the typical actions performed with this plugin will be described. This will be made following both case studies displayed in the visualization paper. All the necessary files are available in the "materials" folder, which are available by unzipping the file in the following link: http://www.optflux.org/suppmaterial/visualization/materials.zip

Basic information about the functionalities of the plugin can be found in the Optflux's wiki page: http://darwin.di.uminho.pt/optfluxwiki/index.php/OptFlux3:METAVIZ

Note that, along with all the files necessary for the tutorial, the folder will also contain project files for both case studies that can be loaded into OptFlux, with the layouts and simulation results included as objects in the clipboard. This will allow immediate testing of both cases shown in the paper, by simply importing the projects.

Usage example: succinate production with *E. coli*

Import layouts and overlap simulation results

In this first case study, the aim is the visualization of genetic modifications to *E. coli* that improve the production of succinate. The following steps need to be followed:

1) Create a new project in Optflux using the *E. coli* model: SBML file "*iJR904.xml*" available in the *materials/models* folder. More information on how to create a project and load a model is available at: http://darwin.di.uminho.pt/optfluxwiki/index.php/OptFlux3:CORE:NewProject

2) To import the used layout, select the option *"File -> Import -> Import Layout"*

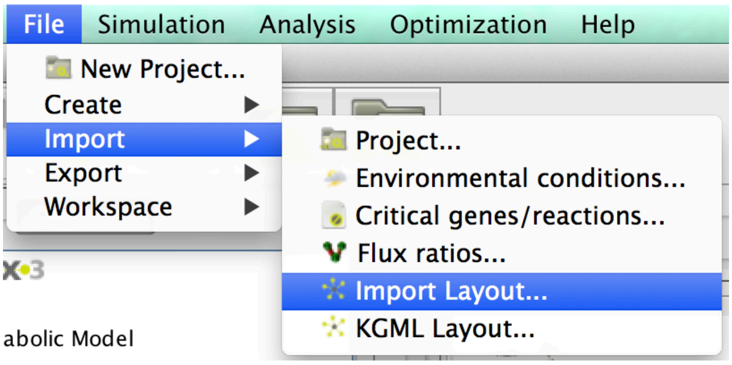

Figure 1 - Import layout menu.

The interface depicted below will appear, where it is possible to select the layout and mapping files for the entities of the layout. To learn more about this, check: http://darwin.di.uminho.pt/optfluxwiki/index.php/Layout:Import

In this case, only the XGMML layout file will be necessary, which is available at *"materials/layouts/ijr/ijr.xgmml".*

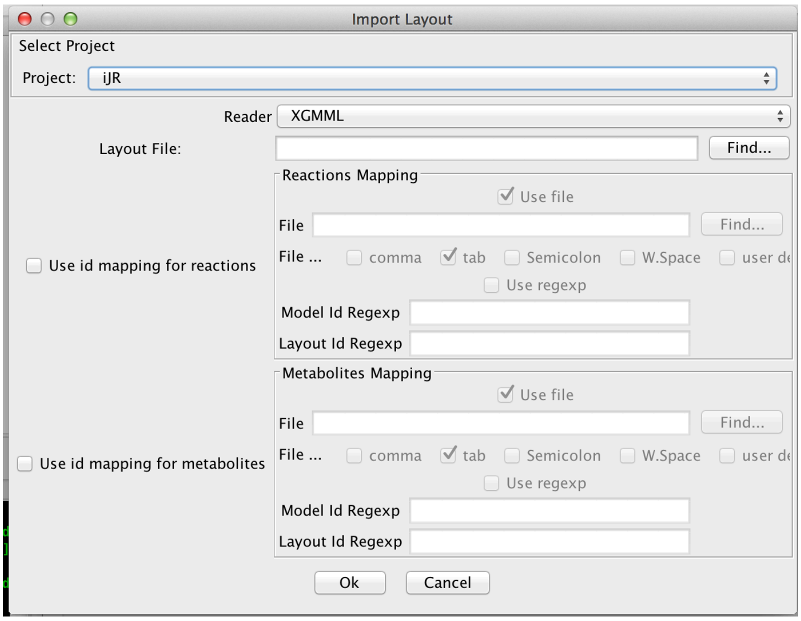

Figure 2: Import layout interface. In this menu, it is possible to specify the type of layout file, and mapping files for the entities in the layout.

After loading the layout, it can be accessed through the clipboard, by clicking the respective item under the category "Layout".

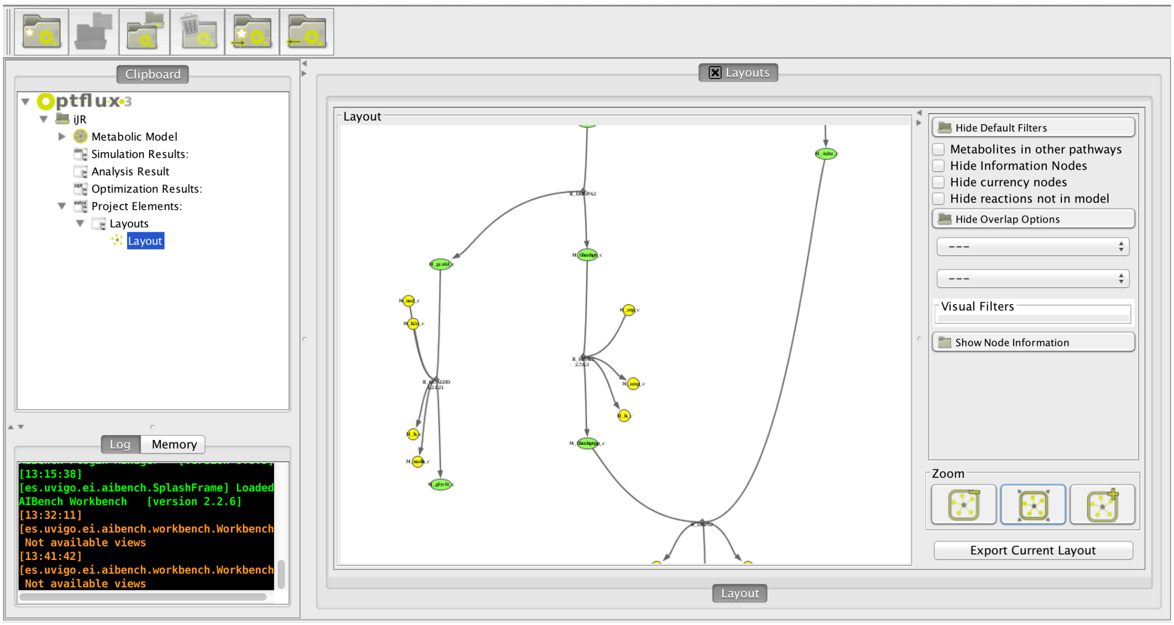

Figure 3 - Visualization interface in Optflux.

3) The next step requires performing a knock-out mutant simulation using Optflux. More details on how to perform this operation can be obtained in the wiki (http://darwin.di.uminho.pt/optfluxwiki/index.php/OptFlux3:SIM:Knockouts). Accessing the *"Simulation -> Knockout -> Reaction"* menu, it is possible to select a list of knockouts and to perform the simulation. For this specific simulation select the following knockouts: R_GHMT2, R_SUCD1i, THD2 and R_TKT1 as shown in the picture below:

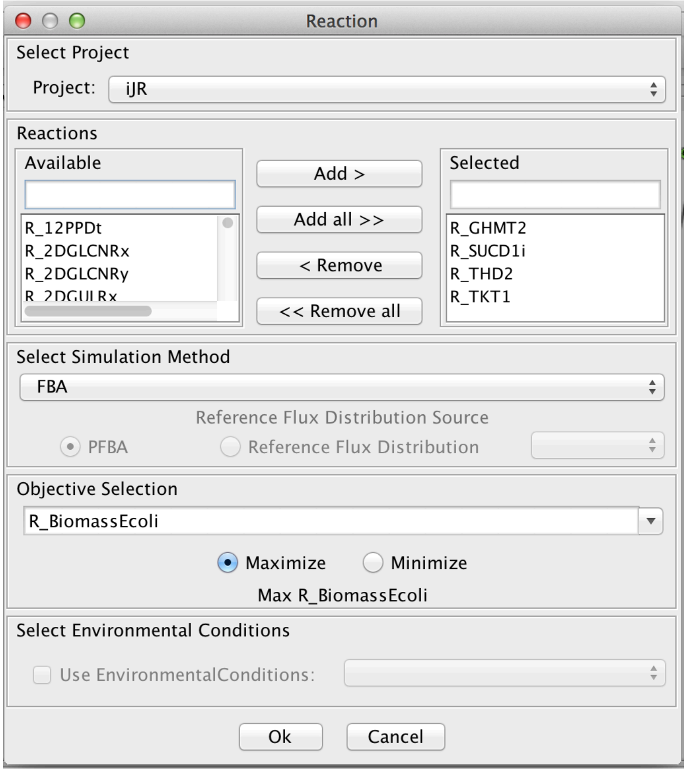

Figure 4 - Knockout mutant simulation menu interface.

Press OK and the simulation result will be available in the clipboard.

4) In the right panel of the visualization plugin interface, there is a section for configuring the overlaps. As soon as any simulation is performed, an item is added to the overlap panel. From there, an user can select which simulation she/he wants to be overlaid over the visualized layout.

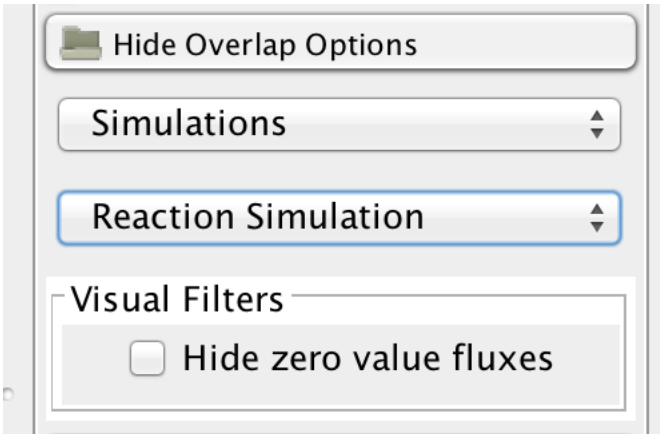

Figure 5 - Overlap panel in the visualization interface.

Some overlaps also have visual filters associated with them. If the user ticks the "Hide zero value fluxes" filter, all the fluxes from this specific simulation that do not carry any flux will be invisible. From this point, and overlaying the previously performed simulation, it is possible to visualize the different fluxes and genetic modifications in the network.

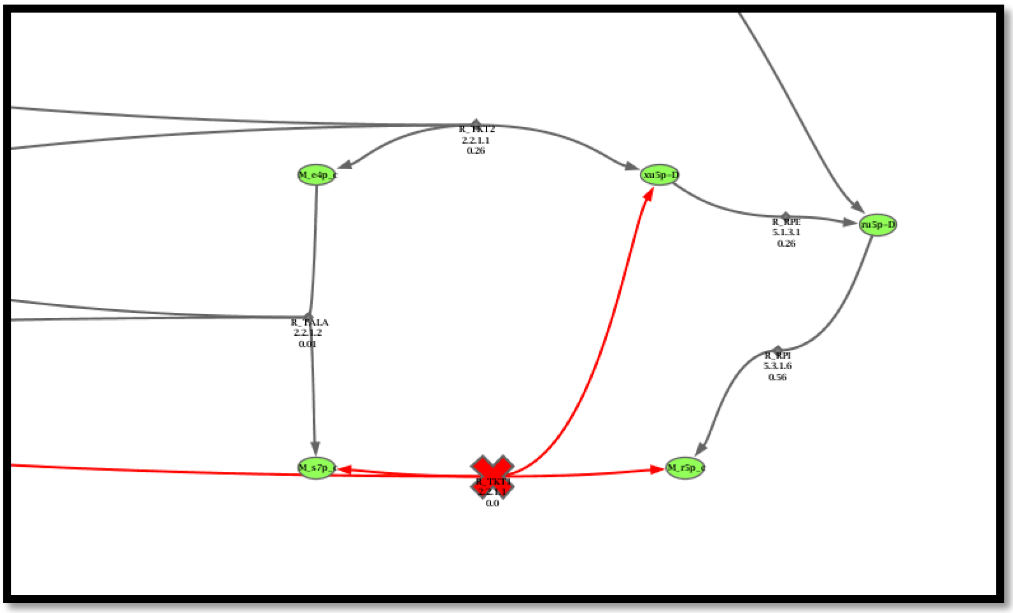

Figure 6 - Knockout example in the network overlapped with the previously performed simulation.

Usage example: glycine production with *E. coli*

1) The first step is similar to the previous case. Create a new project in Optflux, using the *iAD1260* model, whose SBML file is located at "materials/models/iAF1260.xml".

2) Due to the model size, it is preferable if the layouts represent parts of the model, e.g. specific pathways. For this case study, four layouts will be used. The different XGMML files for these pathways are available in the folder "materials/layouts/pathways/". Similarly to the previous case, load the four files XGMML pathway layouts (central metabolism, alternate carbon sources, co-factor biosynthesis and nucleotide metabolism). It is a good practice to change the name of the loaded layout in the clipboard (Right click -> rename element) for better comprehension. At the end of these operations, the clipboard should look like the figure below.

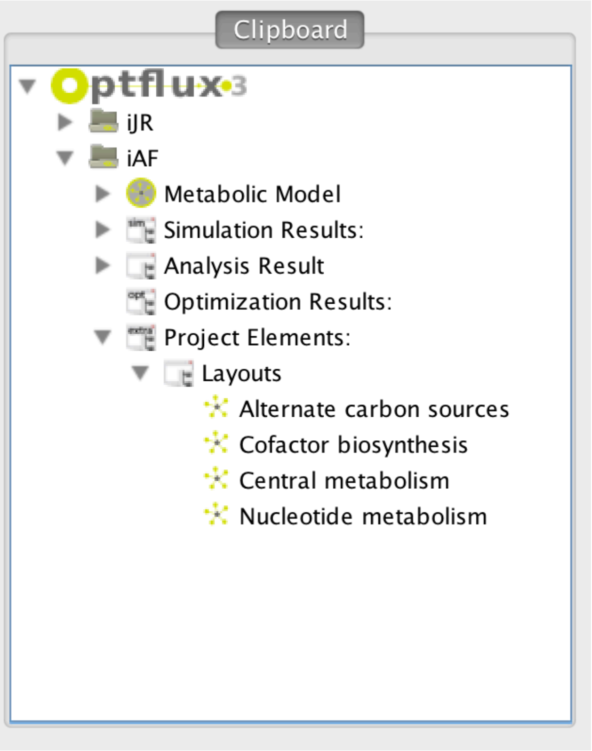

Figure 7 - Clipboard after importing several layouts.

3) The next step consists in performing the simulations. First, perform a wild type simulation, available from the menu "Simulation -> Wild type". After this, and similarly to the previous case, perform a knockout simulation, using the following knockouts: R_ICL, R_GLYCL, R_PPC and R_GART.
Finally, and to demonstrate the analytical capabilities of the visualization plugin, create a comparison between the wild type simulation and the knockout simulation, by accessing the menu "Analysis -> Simulation Comparison".

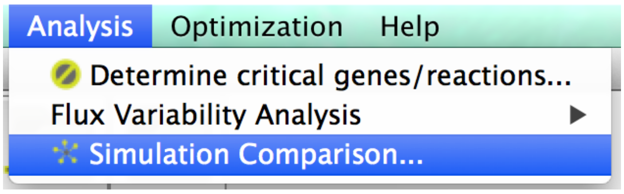

Figure 8 - Simulation comparison under the Analysis menu.

Then, select the simulations you want to compare (in this case only the 2 simulations performed are available).

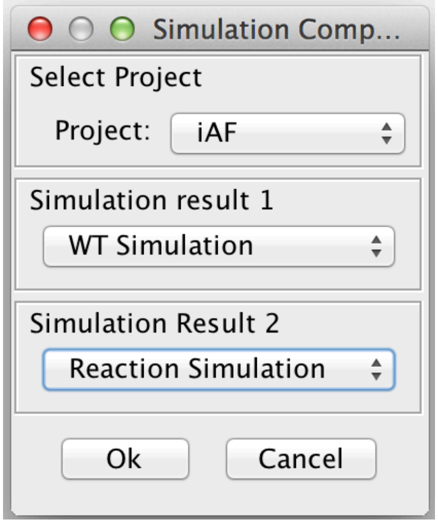

Figure 9 - Simulation comparison menu.

4) When the comparison is performed, a new overlap will be added to the visualization interface, accessible through the overlap options. This overlap will be under the category "Comparisons".

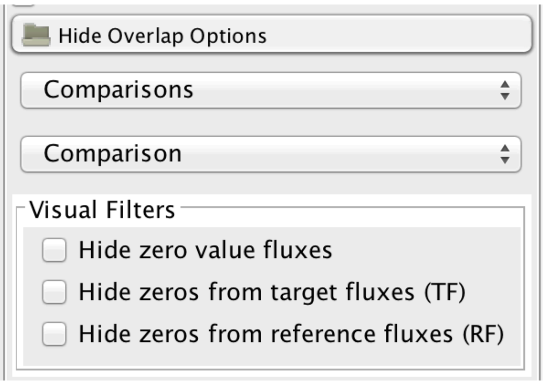

Figure 10 - Overlap options panel showing a simulation comparison and associated filters.

By choosing this overlap, some new filters also become available. In this case, the "Hide zero value fluxes" hides all reactions that have a zero flux in both simulations. The other two filters hide zero values specific to each of the simulations, allowing the user to visualize different scenarios (for instance, if the 3 filters are selected, only reactions with fluxes on both simulations will be displayed).

Below, we show some screenshots of the simulation comparison. Additional information can be obtained in the OptFlux's wiki page: http://darwin.di.uminho.pt/optfluxwiki/index.php/Layout:Overlaps.

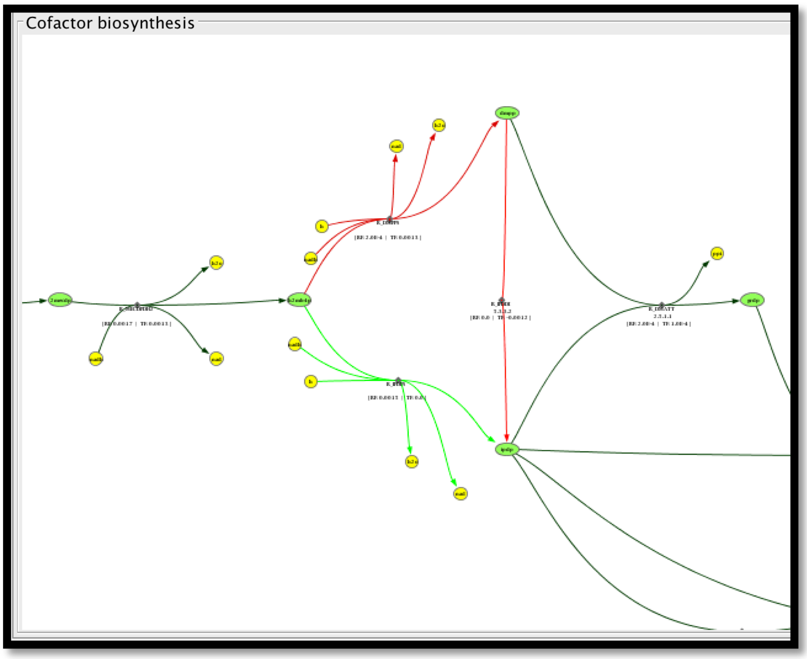

Figure 11 - Part of the cofactor biosynthesis pathway.

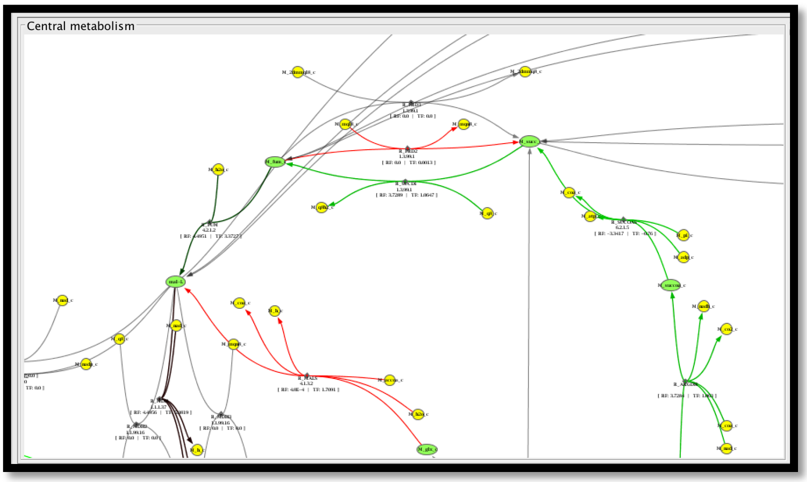

Figure 12 - Part of the central metabolism pathway.

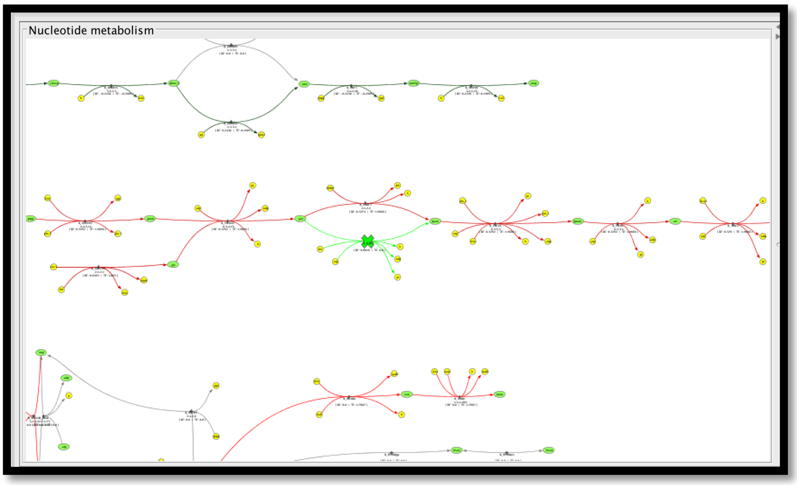

Figure 13 - Part of the nucleotide metabolism pathway.

Visualization preferences

Another interesting component of the visualization plugin that is worthwhile to mention are the "Visualization preferences" that can be accessed through the menu "Help -> Preferences".

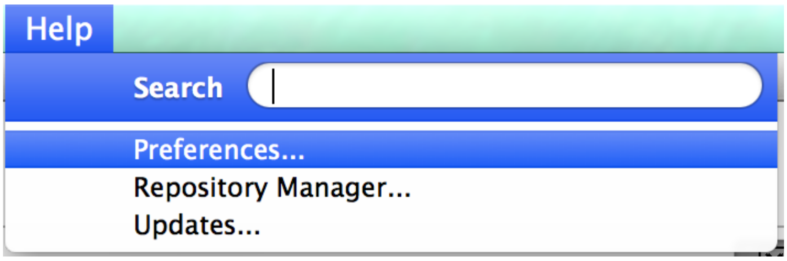

Figure 14 - Preferences in the Help menu.

Clicking in the preferences will show a tree, where it is possible to change some of Optflux's default definitions for several components. The visualization preferences are under the branch "Visualization".

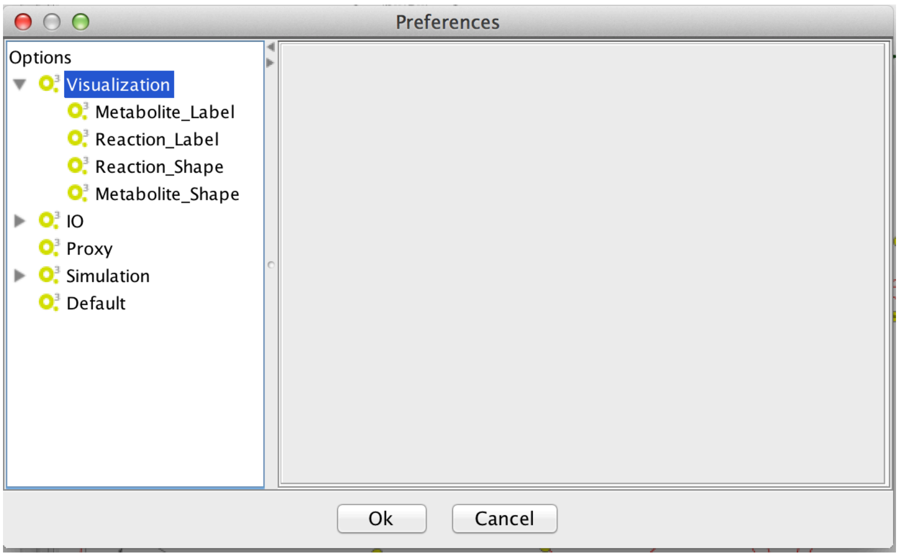

Figure 15 - Preferences menu interface. The visualization options are shown below the branch Visualization.

Here, it is possible to edit two main components of the visualization. One of them is the configuration of the *labels*. It is possible to edit the font and color of the labels, both for the metabolite and reaction nodes. The most interesting point is that it is also possible to edit the information displayed in these labels.

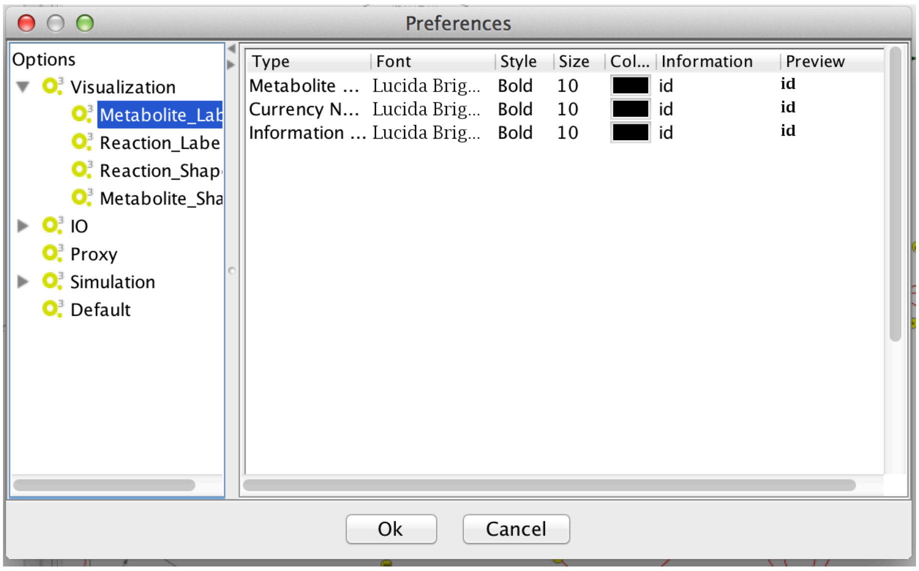

Figure 16 - Metabolite labels configuration.

In the interface above, it is possible to select which of the metabolite nodes' labels the user wants to edit. It is possible to change the font, style, size and color of these labels, as well as the content by clicking in the field "Information". These fields are also available for the reactions' labels. Indeed, in the example below the reactions' labels content was modified.

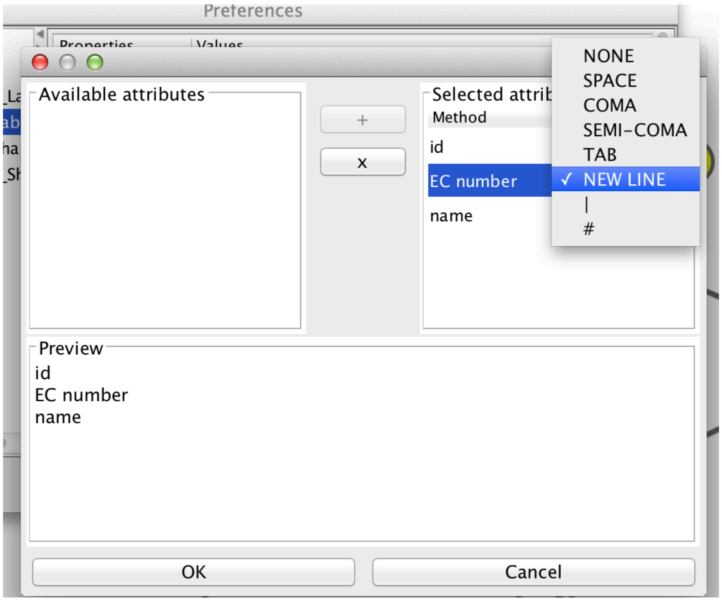

Figure 17 - Editing the label content of the reaction nodes.

For the reaction nodes, a list of available attributes is displayed in the left box of the menu displayed in the image above. The right list contains the selected attributes to display in the node. It is also possible to define the separators for these attributes to be displayed, and to preview of the result in the box below. In this case, all the available attributes for this model were selected, a separated by NEW LINE. The final result is displayed below.

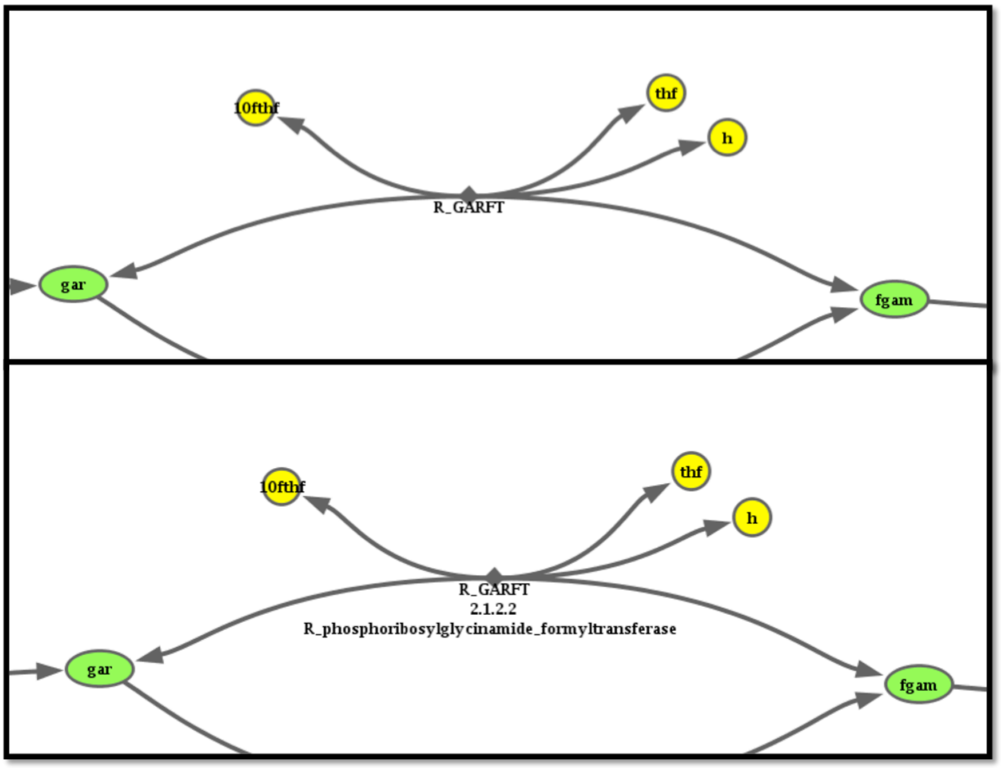

Figure 18 - Reaction labels before and after altering the content. The image above shows only the reaction identifier, while the other displays the identifier, EC number and name separated by new lines.

The other set of configurable options includes the shapes and colors of the nodes. For both reaction and metabolite nodes, it is possible to change the shape of the node from a list, the color of both, the fill and the stroke, and also the stroke thickness.

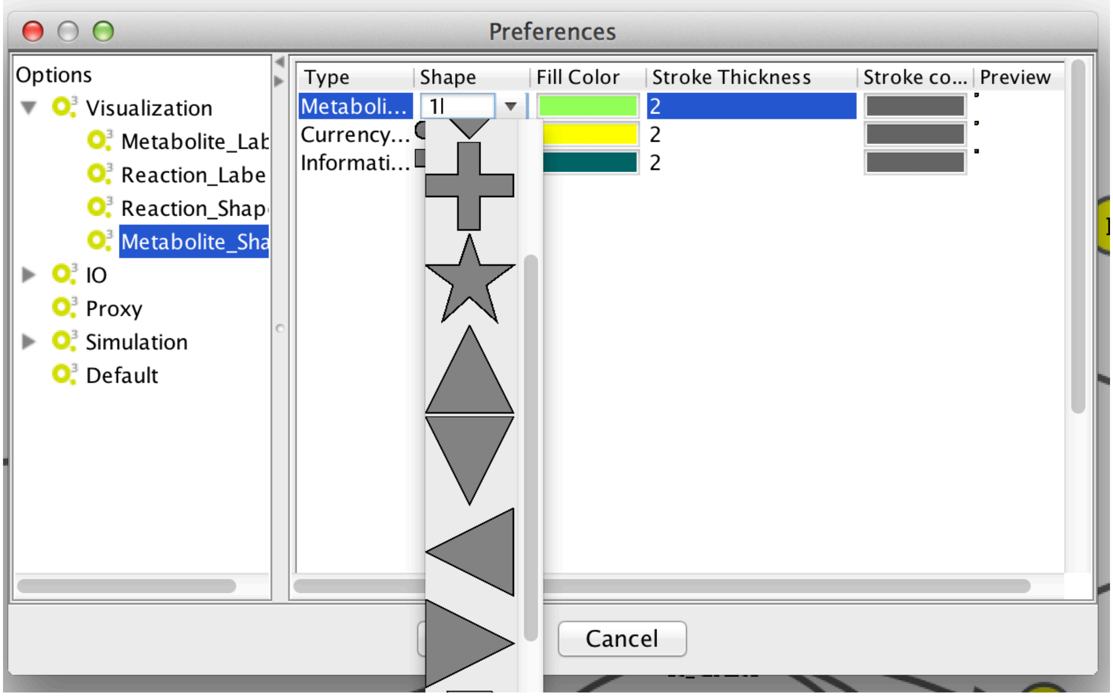

Figure 19 - Configuring the shape of a type of node.
